# The Role of Stacking Interactions in Complexes of Proteins with Adenine and Guanine Fragments of Ligands

**Published:** 2009-04

**Authors:** T. V. Pyrkov, D. V. Pyrkova, E. D. Balitskaya, R. G. Efremov

**Affiliations:** 1 Shemyakin and Ovchinnikov Institute of Bioorganic Chemistry, Russian Academy of Sciences, ul. Miklukho-Maklaya 16/10, Moscow, 117997, Russia;; 2 Moscow Institute of Physics and Technology (State University), Institutskii per., 9, Dolgoprudny, Moscow oblast, 141700, Russia;; 3 Moscow State University, Moscow, 119991, Russia

## INTRODUCTION

The biological function of proteins is closely connected to interactions with their ligands and substrates. Proteins acting as receptors and enzymes bind these small molecules. Knowledge of the molecular mechanisms of protein-ligand interactions, particularly in the spatial structure of the protein-ligand complex, is a prerequisite for understanding the structure-functional properties of proteins and their role in biochemical pathways in the living cell. The availability of such a structure serves as a basis for rational drug design projects and greatly assists the search for new inhibitors (the ligands of certain protein-targets in an organism).

Experimental tools for determining the spatial structure of proteins and their complexes with ligands (such as X-ray crystallography or NMR spectroscopy) have particular limitations. Even if the structure of a protein is available, determining the structure of its complex with ligands may be experimentally demanding. Problems with purification and crystallization become especially difficult in studies of transmembrane proteins, which include a biologically important class of G-protein coupled receptors. However, recent successes in determining the structure of beta-adrenergic and adenosine receptors [1] are cause for optimism.

The technical difficulties restraining experimental methods stimulated computational molecular modeling. One of them (molecular docking) is a method aimed at predicting the spatial structure of a protein-ligand complex by docking a ligand molecule into the known atomic-resolution structure of a protein-binding site and estimating the reliability of the results. Nowadays, molecular docking has become an integral part of both fundamental studies aimed at understanding the structure-functional role of protein amino acids and applied drug-design programs [2, 3].

Docking approaches are further improved by implementing new algorithms of the conformational search and new scoring functions (methods to estimate the free energy of ligand binding). Scoring functions may include either components of molecular mechanics force fields [2] or empirical terms, e.g. hydrogen bonds described by their geometrical parameters [4]. In this work we studied stacking interactions, which usually are not properly taken into account in widely used scoring functions.

## The Parameters of Stacking Interactions

**Fig. 1.**
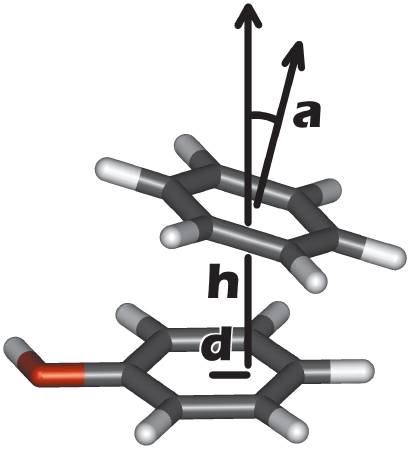
Geometrical parameters used to describe a stacking contact between two aromatic rings. Displacement (d) and height (h) are calculated for the center of one aromatic ring relative to another ring's plane. Angle α is calculated as the angle between the normal vectors of both rings.

Of all the various types of interactions in biomolecular complexes (such as hydrogen bonds, salt bridges, etc.), the stacking of aromatic substances deserves special attention. Most drugs include aromatic fragments in their chemical structure, and stacking often plays a notable role in their recognition by protein-targets. We have recently shown that an explicit account of stacking in scoring functions increases the efficiency of ATP docking [5]. The aromatic interactions were identified by the mutual orientation of two cycles described by geometrical parameters: the height h and displacement d of one cycle relative to the other, and the angle α between their planes (Fig. 1). 

However, the range of these parameters, which corresponds to the presence or absence of a stacking contact, is still not very well defined and usually taken as arbitrary [6, 7]. Defining it more accurately would assist in developing more efficient scoring functions and should increase the prediction quality of the spatial structures of protein-ligand complexes by molecular modeling methods. With this aim in view, we performed an analysis of the spatial structures of protein-ligand complexes determined experimentally with atomic resolution where ligands contained adenine or guanine as a substructure.

One well-known example of stacking interactions is the parallel packing of purine and pyrimidine nucleobases in DNA [8, 9]. Some aromatic compounds tend to orient perpendicular to each other (T-shaped stacking), as has been shown for amino acids in proteins [7, 10] and for model systems of carbon aromatic cycles (benzene and naphtalene) [11–14]. Besides, such compounds participate in cation-π interactions, where a positively charged group interacts with the negatively charged cloud of aromatic π-electrons [15–17].

**Fig. 2.**
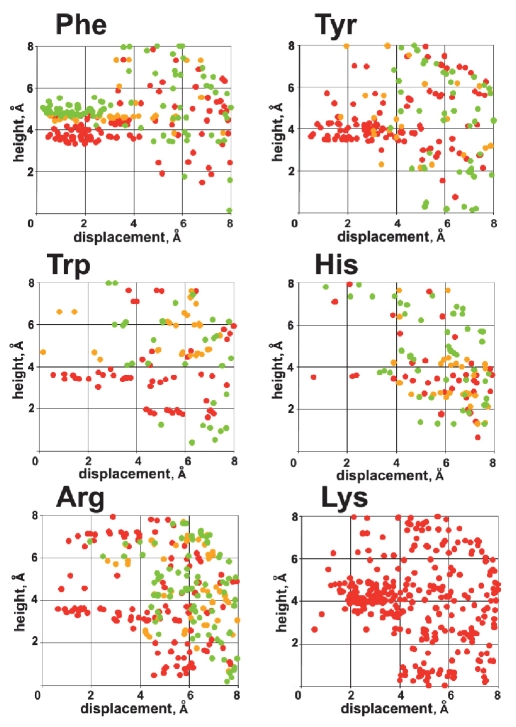
The distribution of aromatic rings and positively charged side chain groups of amino acids around the guanine moiety of various ligands in complexes with protein receptors. The color red corresponds to cos^2^α = 0.6 – 1.0 (parallel orientation), green corresponds to cos^2^α = 0.0 – 0.4 (T-shaped orientation), and yellow corresponds to intermediate geometry. Here, α is the angle between the planes of both rings. For Lys this value is not defined.

Taking all that into account, we analyzed the distribution of geometrical parameters h, d, and α for contacts of adenine and guanine moieties of ligands with the aromatic side chains of receptor amino acids Phe, Tyr, Trp, and His, as well as with the positively charged guanidine group of Arg and amino group of Lys. The results obtained for guanine are presented in Fig. 2.

It can be seen that two distinct orientations are typical for Phe: parallel and perpendicular to the guanine plane (Fig. 2, shown in red and green, respectively). The displacement d lies in the same range 0–3 Å for both types of contacts. Meanwhile, they clearly differ in the value of height h, which is less than 4.5 for parallel Å and less than 5.5 Å for perpendicular orientation. Similar distributions were obtained for Tyr, Trp, and His, though the data are scarcer in these cases. However, the T-shaped contact is not as typical for Tyr, Trp, and His as it is for Phe.

Interestingly, the distribution for the planar guanidine group of Arg very much resembles that of Tyr, Trp, and His, where the orientation parallel to guanine is predominant. For the amino group of the Lys side chain, two modes of contacts with guanine were observed: above the plane (cation-π interaction) and in the plane. The latter corresponds to the formation of a hydrogen bond to the heteroatoms of the guanine ring.

Distributions for adenine are similar to those obtained for guanine (data not shown). The results of the presented analysis may be used in developing scoring criteria and applied to rescoring the results of docking or even during the docking procedure.

## Guanine-Specific Scoring Function

We demonstrated the efficiency of an explicit account of stacking interactions in a scoring function along with reranking the results of GTP docking to the 14 different proteins that bind this ligand. All structures of GTP-protein complexes, which were generated with the docking procedure, were labeled as either correct or misleading by the value of root-mean-square deviation (rmsd) of the guanine atoms from the reference X-ray structure (see Methods). To estimate the validity of a docking pose, we constructed a number of scoring criteria in the form of a linear combination of the interaction terms. To do that, all GTP-protein complexes were divided into two equal groups: the training and the test sets. The weighting coefficients for these terms were fitted by the linear regression procedure to the binary function, which took on the value of 1 for the correct docking poses and 0 for the seven complexes of the training set. To test the robustness of the new scoring functions, the leave-one-out cross-validation procedure was performed. The relative error of all weighting coefficients of interaction terms was less than 30%, thus indicating the reliability of the results.

New scoring functions were used to analyze the GTP docking poses generated for each complex. The efficiency of the goldscore [18] function implemented in the docking algorithm achieved approximately 50%; it ranked correct poses at the top for only four and three complexes out of seven for the training and test sets, respectively (Table 1). Ranking by the value of Tstacking, which describes stacking contacts, was better than by goldscore; their combination (SF1) yields even better results. A similar effect was observed when the term Tstacking was added to the criterion based on lipophilic contacts and hydrogen bonds between the protein and the guanine moiety of the ligand (SF2 and SF3, Table 1). 

**Table 1:** Ranking of the results of GTP docking.

Ranking method	Training set, 7 complexes		Test set, 7 complexes	
Number of complexes for which correct pose was ranked at top	Average best rank of correct pose	Number of complexes for which correct pose was ranked at top	Average best rank of correct pose
goldscore	4	4.7	3	12.1
T_stack_	5	2.3	3	7.0
*SF1* = Р 1.3 + 0.21 × T_stack_ + 0.016 × *goldscore*	5	1.7	4	6.9
*SF2* = 0.06 + 0.007 × T_lipophilic_+ 0.43 × T_h-bond_	5	2.3	4	6.9
*SF3* = 0.05 + 0.004 × T_lipophilic_+ 0.39 × T_h-bond_ + 0.22 × T_stack_	6	2.0	5	6.4

**Fig. 3.**
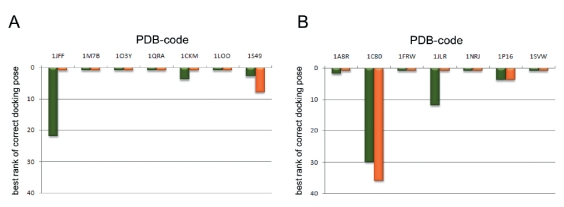
Efficiency of ranking the docking poses for GTP-protein complexes by the SF3 (red) and by goldscore (green) for the training (A) and the test (B) sets. The best rank of a correct GTP pose is shown.

Of the proposed scores, the SF3 is the most efficient. The number of complexes for which the correct pose was ranked at the top by SF3 considerably exceeds that of goldscore. Also, the average best rank of the correct docking pose improves; i.e. the quality of ranking increases uniformly for all complexes. This can also be seen from the results of ranking for each complex (Fig. 3). 

## CONCLUSIONS

The analysis of structural data for complexes of proteins with ligands containing adenine or guanine moiety yielded a more accurate definition of the geometrical parameters of stacking interactions with aromatic side chains of receptor amino acids. Reranking the results of GTP docking demonstrated that an explicit account of stacking in scoring criteria provides a more efficient estimation of the reliability of the structure of the protein-ligand complex predicted with molecular modeling approaches. The obtained results can be further applied to a broader class of nucleobase-containing ligands.

## METHODS

The structures of complexes of proteins with adenine- and guanine-containing ligands were taken from the Brookhaven Protein Data Bank (PDB) [19]. The PDBlig web server [20] was used to identify those PDB entries that contain a ligand with a purine nucleobase (adenine or guanine as a substructure). Structures with modified nucleobases and entries that contain nucleic acids other than simple nucleotides or nucleosides were omitted. Finally, to reduce the redundancy of the set of protein-ligand complexes, a multiple sequence alignment was carried out using the Clustalw program [21]. After that, all complexes with the same ligand were clustered according to similarity in a protein sequence and the structure with the best resolution from each cluster was retained.

GTP docking was performed using the GOLD [18] program with the goldscore scoring function. The parameters of the docking procedure were taken as default. For each complex, 60 docking poses were generated. An rmsd cut-off 2.5 Å over the coordinates of guanine atoms was used to judge whether a pose was correct or misleading.

The surface area of the hydrophobic GTP-protein contact (as a measure of hydrophobic interactions, Tlipophilic) was calculated based on the concept of Molecular Hydrophobicity Potential (MHP) using the PLATINUM web server [22]. A MHP-scale shift of +0.2 was applied to the ligand surface to achieve a more realistic distribution of the hydrophobic/hydrophilic properties of GTP.

The term Th-bond is a binary function that takes on the value of 1 when a hydrogen bond network of guanine and a particular motif is formed; if not, it takes on a value of 0. Such motifs were hydrogen bonds between the guanine atoms N1, N2, and O6 and residues i, i, i, or i, i, i-2. 
